# A Pontryagin class obstruction for purely electric and purely magnetic Weyl curvature tensors

**DOI:** 10.1007/s11005-026-02152-x

**Published:** 2026-09-17

**Authors:** Thijs de Kok

**Affiliations:** Department of Mathematics, IMAPP, Radboud University, PO Box 9010, Postvak 59, 6500 Nijmegen, Gelderland The Netherlands

**Keywords:** Pontryagin class, Algebraic curvature tensor, Weyl curvature tensor, Purely electric/magnetic, Petrov types, Umbilic hypersurfaces, 83C20, 57R20, 53C50, 53C12, 53C18

## Abstract

Do all manifolds that admit Lorentzian metrics also admit such metrics that have a purely electric (PE) or purely magnetic (PM) Weyl curvature tensor? To (partially) answer this question, we show that for all algebraic curvature tensors on a 4*k*-dimensional scalar product space that are even or odd under the action of a orientation-reversing isometry, the products of Pontryagin forms that land in the top-degree exterior power of the dual vector space vanish. We use this to derive the vanishing of all products of Pontryagin classes that land in the top-degree de Rham cohomology of a 4*k*-dimensional pseudo-Riemannian manifold with a PE or PM Riemann or Weyl curvature tensor. For compact manifolds, this gives nontrivial cohomological obstructions to the existence of such pseudo-Riemannian metrics with globally PE or PM Riemann or Weyl curvature tensors. These obstructions can be linked to the existence of Lorentzian metrics of several Petrov subtypes, which play an important role in classifying exact solutions to the Einstein equations. Moreover, they can be applied to foliations by nondegenerate umbilic hypersurfaces, which may appear as time slices of spacetimes.

## Introduction

One of the big efforts in general relativity is finding and classifying exact solutions to the Einstein equations. By now, a vast amount of literature is available on this topic. See, e.g., the book by Stephani et al [49], that gives a survey on all exact solutions known up to 1999. A major tool for classifying exact solutions is by posing conditions on the structure of their curvature tensors. One approach is via the splitting of the Riemann/Weyl curvature tensor of a 4-dimensional Lorentzian manifold satisfying the vacuum Einstein equations into an “electric” and “magnetic” part, based on a fixed timelike unit vector field. This was introduced already in the 1950 s by Matte [37, §4]. The terminology is motivated by the observation that the second Bianchi identity becomes a Maxwell-like equation with source terms [37, §5].

On a 4-dimensional oriented Lorentzian manifold, this splitting is performed [49, Ch. 3.5] by first introducing the complex-valued *self-dual Weyl curvature tensor* $\widetilde{W}$ with componentswhere ε is the totally anti-symmetric Levi-Civita symbol satisfying  with respect to a local oriented orthonormal basis . Choosing a unit timelike vector field *T* on *M*, we can then define the tensor fields *E* and *B* aswhich are the *electric* and *magnetic* parts of the Weyl curvature tensor, respectively. The electric part *E* captures gravitational effects that resemble tidal forces from Newtonian mechanics, whereas the magnetic part *B* has no Newtonian counterpart [18, p. 607–608].

The Weyl curvature tensor is *purely electric (PE)* at p∈M if its magnetic part *B* vanishes and *purely magnetic (PM)* if its electric part *E* vanishes. Note that this is a pointwise property. PE spacetimes arise naturally in studying general relativity: All spacetimes with a unit timelike congruence that is shear-free and irrotational have PE Weyl curvature tensors with respect to the congruence [53]. This includes static spacetimes [49, Ch. 6.2], warped products of the form $\mathbb {R}\times _f\Sigma$ (this follows from a variation of [40, Prop. 42] for the Weyl curvature tensor) and spacetimes with irrotational perfect fluids [4, 5]. On the contrary, some examples of PM spacetimes are known [1, 31, 32] but PM spacetimes are rather elusive in general and do not arise naturally. For example, Van den Bergh [54] showed that there are no PM vacuum solutions to the Einstein equations and Lesame [30] showed that there are no irrotational dust solutions with PM Weyl curvature tensors. More generally, Maartens, Lesame and Ellis [33] showed that metrics with PM Weyl curvature tensors are inconsistent with the Einstein equations in general.

A classical result is that a manifold *M* admits a Lorentz metric if and only if it is either noncompact or is compact and has Euler-characteristic equal to 0. A natural follow-up question to ask is whether a manifold *M* that admits Lorentzian (or in general pseudo-Riemannian) metrics also admits such metrics with PE or PM Weyl curvature tensors. For 4-dimensional Lorentzian manifolds, the property of having a PE or PM Weyl curvature tensor was found to be closely related to the Petrov type of the Weyl curvature tensor. In particular, the Weyl curvature tensor *W* of a 4-dimensional Lorentzian manifold *M* is PE/PM at p∈M if it has Petrov type *O*, *I* or *D* with real/imaginary eigenvalues [25, Rem. 3.8] [38, p. 1556] [53, 55]. Furthermore, Avez [3] showed in the 1970 s that geometric properties of the Weyl curvature tensor can be related to the smooth and topological structure of the manifold via the Pontryagin forms and classes. The *Pontryagin forms* of a pseudo-Riemannian manifold (*M*, *g*) are differential forms

$$ \varpi _k(M) \in \Omega ^{4k}(M), $$

which are constructed from the Riemann curvature tensor of the Levi-Civita connection of (*M*, *g*). The Pontryagin forms are closed differential forms and therefore define classes in the de Rham cohomology of the manifold *M* [39, p. 296]. The resulting *Pontryagin classes*

$$ p_k(M):=[\varpi _k(M)] \in H^{4k}_{dR}(M) $$

do not depend on the chosen metric *g* and are proper invariants of *M* [39, p. 298]. We refer to Sect. 2.3 for a precise introduction of the Pontryagin forms and classes. Clearly, the Pontryagin form $\varpi _k(M)$ and hence the Pontryagin class $p_k(M)$ vanish if 4*k* is greater than the dimension of *M*. In particular, for 4-dimensional manifolds *M*, only the first Pontryagin form and class may be nontrivial. Avez showed that if *M* admits a locally conformally flat metric—that is, with vanishing Weyl curvature tensor *W*, which is by definition of Petrov type *O*—then the first Pontryagin form $\varpi _1(M)$ (and therefore also $p_1(M)$) vanishes. Later, Zund [58] and Porter and Thompson [45] extended the result by Avez to other Petrov types, including type *D* with real or imaginary eigenvalues, which are PE or PM. This shows that on 4-dimensional Lorentzian manifolds, the Pontryagin class $p_1(M)$ may function as an obstruction to the existence of Lorentzian metrics with PE or PM type *D* Weyl curvature tensors. Recently, Hervik, Ortaggio and Wylleman [25] observed that for the Weyl curvature tensor, being PE or PM with respect to a timelike unit vector *T* is equivalent to being even or odd under the action of the orthogonal reflection along the hyperplane $T^{\bot }$. Using their characterization, the electric–magnetic splitting naturally extends to algebraic curvature tensors on vector spaces of arbitrary signature and dimension and can be performed for all vectors *T* that are not null.

In this paper, we discuss in what way the Pontryagin classes of a manifold *M* give an obstruction to the existence of PE or PM Weyl curvature tensors in the sense of Hervik et al. In Sect. 3.2, we will obtain the following precise restrictions on the Pontryagin forms and classes of a manifold admitting pseudo-Riemannian metrics with PE or PM Weyl curvature tensors. To the best of our knowledge, this is the first result relating the property of being PE or PM to the Pontryagin classes of the manifold directly. For the precise statement of the presented results, we refer to the corresponding results in the text.

### Theorem 1.1

(Theorem 3.10). Let (*M*, *g*) be a 4*k*-dimensional pseudo-Riemannian manifold and suppose that the Weyl curvature tensor *W* is PE or PM at each point. Then for all multi-indices $\alpha = (\alpha _1, \ldots , \alpha _l) \in \mathbb {Z}_{> 0}^l$ with $\alpha _1+\cdots +\alpha _l = k$, the 4*k*-forms

$$\begin{aligned} \varpi _{\alpha }(W) := \varpi _{\alpha _1}(W)\wedge \cdots \wedge \varpi _{\alpha _l}(W) = 0 \in \Omega ^{4k}(M). \end{aligned}$$

In particular, the class

$$\begin{aligned} p_{\alpha }(M):= p_{\alpha _1}(M)\smile \cdots \smile p_{\alpha _l}(M) = 0 \in H^{4k}_{dR}(M). \end{aligned}$$

Our proof of Theorem 1.1 is purely algebraic in the sense that it only depends on the algebraic symmetries of the Weyl curvature tensor and the algebraic restrictions that being PE or PM yield for the Weyl curvature tensor. Therefore, we prove Theorem 1.1 generally for all algebraic curvature tensors that satisfy a PE- or PM-like algebraic condition. We refer to Sect. 3.2 for the general setup. The main idea of the proof is to show that if an algebraic curvature tensor *C* on a 4*k*-dimensional scalar product space (*V*, *g*) is even or odd under the action of a linear isometry of *g*, then the maps induced by the isometry on the exterior powers of the dual space $V^*$ fix the Pontryagin forms of the algebraic curvature tensor. Consequently, also products of such Pontryagin forms are fixed by these maps. The result then follows from the observation that if the determinant of such isometry is -1, then the only element in $\wedge ^{4k}V^*$ that is fixed is 0.

Using that the Weyl curvature tensor of a 4-dimensional Lorentzian manifold of Petrov type *I* with real or purely imaginary eigenvalues is PE or PM [38, p. 1556], we can extend the results of Avez, Zund, Porter and Thompson and also add Petrov type *I* with real or imaginary eigenvalues to the list of Petrov (sub)types that guarantee the vanishing of the first Pontryagin class $p_1(M)$ of the manifold *M*.

### Theorem 1.2

(Theorem 3.17). Let *W* be the Weyl curvature tensor of a 4-dimensional Lorentzian manifold (*M*, *g*). If at all points p∈M the Weyl curvature tensor *W* has Petrov type *I* with real or purely imaginary eigenvalues, then $p_1(M) = 0$.

Finally, we discuss an application of Theorem 1.1 to the existence of foliations by nondegenerate umbilic hypersurfaces. Recall that a nondegenerate hypersurface Σ of a pseudo-Riemannian manifold (*M*, *g*) is *umbilic* if the scalar second fundamental form *h* of the embedding $\Sigma \hookrightarrow M$ is of the form h=fσ, where $f\in C^{\infty }(\Sigma )$ and σ is the induced metric on Σ. Umbilic hypersurfaces are studied both in the setting of Riemannian geometry, see, e.g., [12, 20, 28] for just a few examples, and in the setting of Lorentzian geometry, where umbilic *spacelike* hypersurfaces may appear as time slices of spacetimes [2, 10, 19]. At such a nondegenerate umbilic hypersurface, the Weyl curvature tensor is even with respect to the orthogonal reflection along TΣ [46, §2]. This allows us to apply Theorem 1.1.

### Theorem 1.3

(Theorem 3.23). Let (*M*, *g*) be 4*k*-dimensional pseudo-Riemannian manifold. Let $\Sigma \hookrightarrow M$ be a nondegenerate umbilic hypersurface. Then for all multi-indices $\alpha = (\alpha _1, \ldots , \alpha _l) \in \mathbb {Z}_{> 0}^l$ with $\alpha _1+\cdots +\alpha _l = k$, the 4*k*-forms $\varpi _{\alpha }(W)$ vanish at $\Sigma \subset M$.

In particular, if (*M*, *g*) is foliated by such hypersurfaces, then for all such multi-indices α, the 4*k*-form $\varpi _{\alpha }(W)$ vanishes and therefore $p_{\alpha }(M) = 0$.

When formulated contrapositively, Theorems 1.1, 1.2 and 1.3 pose obstructions to the existence of pseudo-Riemannian metrics with globally PE or PM Weyl curvature tensors, Lorentzian metrics with these Petrov subtypes and pseudo-Riemannian metrics for which *M* is foliated by nondegenerate umbilic hypersurfaces, respectively.

### Outline

The paper is structured as follows. In Sect. 2, we briefly recall the notion of an algebraic curvature tensor on a vector space, how it defines a curvature operator on bivectors and we introduce Thorpe’s higher curvature operators acting on the higher exterior powers of the vector space. We also review how the decomposition of the Riemann curvature tensor into its Weyl, Ricci and scalar components carries over to algebraic curvature tensors. We will conclude Sect. 2 with recalling the construction of the Pontryagin forms and classes of a manifold, which motivates defining the Pontryagin forms of an algebraic curvature tensor. We present two important properties of the Pontryagin forms: the first being that we can express the Pontryagin forms succinctly using the higher curvature operators (Theorem 2.18) and the second being that the Pontryagin forms of an algebraic curvature tensor *C* are equal to the Pontryagin forms of its Weyl curvature tensor $W_C$ (Theorem 2.19). The former is mentioned a few times in the literature, but without proof or specifically for the Riemannian setting. Therefore, we provide a proof of the statement in Appendix A. In Sect. 3.1, we will first discuss the approach to PE and PM curvature tensors by Hervik et al., characterizing PE and PM curvature tensors as the curvature tensors that are even or odd under the action of a reflection in the hyperplane orthogonal to a timelike unit vector. In Sect. 3.2, we prove our main result Theorem 1.1 and give an example of a manifold that admits Lorentzian metrics but none with a PE or PM Weyl curvature tensor. We conclude the paper by discussing resulting obstructions for the existence of some Petrov subtypes (Theorem 1.2) and foliations by nondegenerate umbilic hypersurfaces (Theorem 1.3).

## Preliminaries

In this section, we first recall the notion of an algebraic curvature tensor on a vector space and the associated (higher) curvature operators acting on the even exterior powers of that vector space. In Sect. 2.2, we review the familiar splitting of the Riemann curvature tensor into its Weyl, Ricci and scalar components in the setting of algebraic curvature tensors. Finally, in Sect. 2.3, we conclude by introducing the Pontryagin forms and classes of a manifold.

Unless specified otherwise, we follow the conventions of Lee [29]. Our manifolds are second countable, Hausdorff and $C^{\infty }$. Let *M* be an *n*-dimensional manifold and let *g* be a pseudo-Riemannian metric on *M* with signature (*p*, *q*), where *p* denotes the number of timelike directions.

### Algebraic curvature tensors

Let ∇ be the Levi-Civita connection of *g*. We use the convention of Lee [29] and define the *Riemann (curvature) tensor* for all $U, V, X \in \mathfrak {X}(M)$ by

2.1 $$\begin{aligned} R(U, V)X = [\nabla _U, \nabla _V]X-\nabla _{[U, V]}X, \end{aligned}$$

which is $C^{\infty }(M)$-linear in *U*, *V* and *X* and therefore defines a (1, 3)-tensor field on *M*. Alternatively, we can use the metric *g* to express *R* as a (0, 4)-tensor field *Rm* on *M* defined for all $U, V, X, Y \in \mathfrak {X}(M)$ by

2.2 $$\begin{aligned} Rm(U, V, X, Y) = g(R(U, V)X, Y), \end{aligned}$$

which we also refer to as the Riemann (curvature) tensor. Based on the symmetries of the Riemann tensor, we can introduce the algebraic curvature tensors as the (0, 4)-tensors that satisfy the same algebraic symmetries.

#### Definition 2.1

Let *V* to be a finite-dimensional vector space over $\mathbb {R}$. An *algebraic curvature tensor* on *V* is a (0, 4)-tensor *C* on *V* that for all u,v,x,y∈V satisfies the symmetries:

i) C(u,v,x,y)=-C(v,u,x,y)=-C(u,v,y,x),
ii) C(u,v,x,y)=C(x,y,u,v) and
iii) C(u,v,x,y)+C(x,u,v,y)+C(v,x,u,y)=0.

We denote the vector space of algebraic curvature tensors on *V* by $\mathcal {C}(V)$. An *algebraic curvature tensor field* on a manifold *M* is a (0, 4)-tensor field *C* on *M* such that $C_p$ is an algebraic curvature tensor on $T_pM$ for all p∈M.

We recall the associated curvature operator of an algebraic curvature tensor and its higher analogs. Let (*V*, *g*) be a scalar product space and let $C \in \mathcal {C}(V)$ be an algebraic curvature tensor. Recall that the scalar product *g* on *V* induces a nondegenerate scalar product $\langle \cdot ,\cdot \rangle _g$ on $\wedge ^kV$ that is uniquely defined by

2.3 $$\begin{aligned} \langle v_1\wedge \cdots \wedge v_k, w_1\wedge \cdots \wedge w_k\rangle _g = \det (\{g(v_i, w_j)\}_{1\le i, j\le k}) \end{aligned}$$

for all $v_i, w_j \in V$ and extended bilinearly to all of $\wedge ^k V$ [22, Ch. 4.8, 5.4].

#### Definition 2.2

The *curvature operator* associated with the algebraic curvature tensor *C* is the unique linear operator $\hat{C}$ on $\wedge ^2V$ that is defined by

$$ \langle \hat{C}(u\wedge v), x\wedge y\rangle _g = C(u, v, x, y), $$

for all u,v,x,y∈V.

#### Remark 2.3

In Definition 2.2, we use the opposite sign as in Lee [29, p. 262]. This is chosen so to stay more consistent with sign conventions of other references in later parts of this paper.

The curvature operator has higher analogs, which are linear operators on the vector spaces $\wedge ^{2k}V$ and were introduced by Thorpe in [50]. These are constructed by extending the curvature operator $\hat{C}$ to the vector spaces $\wedge ^{2k}V$ by taking powers under the product of the mixed exterior algebra [22, Ch. 6]. For simplicity, we do not wish to introduce all notation regarding the mixed exterior algebra and follow the construction of the higher curvature operators by Bivens [8].

#### Definition 2.4

Let $A \in \operatorname {End}(\wedge ^k V)$ and $B \in \operatorname {End}(\wedge ^l V)$. We define $A*B\in \operatorname {End}(\wedge ^{k+l} V)$ as the unique linear operator satisfying

$$ (A*B)(x_1\wedge \cdots \wedge x_{k+l}) = \frac{1}{k!l!}\sum _{\sigma \in S_{k+l}}\operatorname {sgn}(\sigma )A(x_{\sigma (1)}\wedge \cdots \wedge x_{\sigma (k)})\wedge B(x_{\sigma (k+1)}\wedge \cdots \wedge x_{\sigma (k+l)}), $$

for all $x_1, \ldots , x_{k+l} \in V$. Here, $S_{k+l}$ denotes the permutation group on k+l elements and $\operatorname {sgn}(\sigma )$ denotes the sign of the permutation σ.

For simplicity, let $[n]=\{1, \ldots , n\}$. If $I =(i_1, \ldots , i_k)\in [n]^k$ is a *multi-index* of elements in [*n*] of *length* *k* and $e = (e_1, \ldots , e_n)$ is a basis for the vector space *V*, then we denote by $e_I = e_{i_1}\wedge \cdots \wedge e_{i_k}$ the iterated wedge product. Recall that $e_I$ vanishes if *I* has a repeated index. Note that the *k*-vectors $e_I$ with increasing multi-index *I* of length *k* form a basis of $\wedge ^kV$ and therefore the collection of all *k*-vectors of the form $e_I$ form a spanning set of $\wedge ^kV$. Expanding a *k*-vector over all such $e_I$ has the advantage that we do not need to control the ordering of the multi-indices *I*, at the cost of working with a nonunique expansion.

Note that the following result is immediate after identifying ∗ as the multiplication on the diagonal subalgebra of the mixed exterior algebra of *V* [22, Ch. 6.2].

#### Lemma 2.5

Let *V* be an *n*-dimensional real vector space and let $0\le k, l \le n$ be arbitrary. Then the operation

$$ *:\operatorname {End}(\wedge ^kV)\times \operatorname {End}(\wedge ^lV)\rightarrow \operatorname {End}(\wedge ^{k+l}V) $$

is associative, commutative and $\mathbb {R}$-bilinear.

If $e = (e_1, \ldots , e_n)$ is a basis of *V* and $A \in \operatorname {End}(\wedge ^k V)$ and $B \in \operatorname {End}(\wedge ^l V)$ are expanded as

$$ A = \sum _{I \in [n]^k}\alpha ^I\otimes e_I \qquad {\text {and}}\qquad B = \sum _{J\in [n]^l}\beta ^J\otimes e_J $$

with $\alpha ^I \in \wedge ^kV^*$ and $\beta ^J \in \wedge ^lV^*$, then

2.4 $$\begin{aligned} A*B = \sum _{\begin{array}{c} I \in [n]^k\\ J\in [n]^l \end{array}}(\alpha ^I\wedge \beta ^J)\otimes (e_I\wedge e_J). \end{aligned}$$

Moreover, for every $\theta \in \operatorname {End}(V)$:

2.5 $$\begin{aligned} (A*B)\circ \wedge ^{k+l}\theta&= (A\circ \wedge ^k\theta )*(B\circ \wedge ^l\theta ), \end{aligned}$$

2.6 $$\begin{aligned} \wedge ^{k+l}\theta \circ (A*B)&= (\wedge ^k\theta \circ A)*(\wedge ^l\theta \circ B). \end{aligned}$$

#### Proof

$\mathbb {R}$-bilinearity of ∗ is clear from $\mathbb {R}$-bilinearity of the wedge product. Equation (2.4) follows directly by writing out A∗B and using the expansion of *A* and *B*. Associativity and commutativity of ∗ are then easily deduced from (2.4). Denote by $\theta ^* \in \operatorname {End}(V^*)$ the induced dual map. Then using (2.4), we see that

$$\begin{aligned} (A*B)\circ \wedge ^{k+l}\theta&= \sum _{\begin{array}{c} I \in [n]^k\\ J\in [n]^l \end{array}}\wedge ^{k+l}\theta ^*(\alpha ^I\wedge \beta ^J)\otimes (e_I\wedge e_J) \\&=\sum _{\begin{array}{c} I \in [n]^k\\ J\in [n]^l \end{array}}(\wedge ^{k}\theta ^*(\alpha ^I)\wedge (\wedge ^{l}\theta ^*(\beta ^J)))\otimes (e_I\wedge e_J) \\&= (A\circ \wedge ^k\theta )*(B\circ \wedge ^l\theta ), \end{aligned}$$

which proves (2.5). Equation (2.6) follows from a similar computation. □

#### Definition 2.6

Let *C* be an algebraic curvature tensor on *V* and denote its curvature operator by $\hat{C}$. Then, we define the *kth curvature operator* associated to *C* as

$$ \hat{C}_{2k} = \hat{C}^{*k} \in \operatorname {End}(\wedge ^{2k}V). $$

#### Remark 2.7

The *k*th curvature operator is labeled with the index 2*k* to keep consistent with conventions in existing literature on higher curvature operators (e.g., [8, 52]).

#### Remark 2.8

(Curvature operators on manifolds). If *C* is an algebraic curvature tensor field on a manifold *M*, applying Definition 2.2 pointwise on each $T_pM$ yields a vector bundle endomorphism

$$ \hat{C}:\wedge ^2TM\rightarrow \wedge ^2TM. $$

Smoothness of $\hat{C}$ can easily be verified by considering its action on bivector fields, see, e.g., [29, p. 262] (note the difference in sign convention, see Remark 2.3). Similarly, applying Definition 2.4 on each $T_pM$ yields a $C^{\infty }(M)$-bilinear operation

$$ *:\operatorname {End}(\wedge ^kTM)\times \operatorname {End}(\wedge ^lTM)\rightarrow \operatorname {End}(\wedge ^{k+l}TM). $$

Therefore, we obtain vector bundle endomorphisms

$$ \hat{C}_{2k} = \hat{C}^{*k}:\wedge ^{2k}TM\rightarrow \wedge ^{2k}TM $$

as higher curvature operators.

### Decomposition of algebraic curvature tensors

The Riemann curvature tensor *Rm* of a pseudo-Riemannian manifold (*M*, *g*) can be decomposed into simpler curvature objects, namely the Ricci curvature, scalar curvature and Weyl curvature tensor of (*M*, *g*) [29, Ch. 7]. In general, we can apply this construction to decompose any algebraic curvature tensor into its Weyl, Ricci and scalar curvature using a choice of scalar product *g*.

Again, let (*V*, *g*) be an *n*-dimensional scalar product space. First, recall that *g* gives rise to traces $\operatorname {tr}^{i, j}_g:T^{(0, k+2)}(V)\rightarrow T^{(0, k)}(V)$ by contracting the *i*th and *j*th entry. For example, if $C \in \mathcal {C}(V)$ is an algebraic curvature tensor and therefore a (0, 4)-tensor on *V*, the contraction of its first and fourth entry is given bywhere  is the inverse metric, satisfying . Denote the symmetric bilinear forms on *V* by  and the trace-free symmetric bilinear forms by . If  are two symmetric bilinear forms on *V*, then their *Kulkarni–Nomizu product* is the (0, 4)-tensorwhich is an algebraic curvature tensor.

#### Definition 2.9

Let *C* be an algebraic curvature tensor. Then, we define its *Ricci curvature* as ${{\,\textrm{Ric}\,}}_C = \operatorname {tr}_g^{1,4}(C) \in \Sigma ^2(V^*)$ and its *scalar curvature* as $S_C = \operatorname {tr}_g({{\,\textrm{Ric}\,}}_C) \in \mathbb {R}$. We also define its *Schouten tensor* as

$$ P_C = \frac{1}{n-2}\left( {{\,\textrm{Ric}\,}}_C-\frac{S_C}{2(n-1)}g\right) \in \Sigma ^2(V^*), $$

and its *Weyl curvature tensor* as

An easy computation shows that the Weyl curvature tensor of any algebraic curvature tensor *C* is trace-free in the sense that $\operatorname {tr}^{1, 4}_g(W_C) = 0$. Moreover, if *C* is trace-free already, then $C = W_C$. Therefore, the following definition makes sense.

#### Definition 2.10

A *Weyl curvature tensor* *W* is an algebraic curvature tensor that satisfies $\operatorname {tr}^{1, 4}_g(W) = 0$. We denote the vector space of Weyl curvature tensors by $\mathcal {W}(V, g) = \mathcal {C}(V)\cap \ker (\operatorname {tr}^{1, 4}_g)$.

#### Remark 2.11

(Curvature objects on manifolds). If instead we have an algebraic curvature tensor field *C* on a manifold (*M*, *g*), then by applying Definition 2.9 pointwise, we obtain smooth symmetric (0, 2)-tensors fields ${{\,\textrm{Ric}\,}}_C$ and $P_C$, a smooth function $S_C$ and an algebraic curvature tensor field $W_C$, which carry the same names as the corresponding objects in Definition 2.9.

We recall the decomposition of an algebraic curvature tensor into its Schouten and Weyl curvature tensor.

#### Proposition 2.12

([29, Prop. 7.24]). For every algebraic curvature tensor *C* on a scalar product space (*V*, *g*) of dimension n≥3, the Weyl curvature tensor $W_C$ is a Weyl curvature tensor in the sense of Definition 2.10, and  is the orthogonal decomposition of *C* corresponding to $\mathcal {C}(V) = \mathcal {W}(V, g)\oplus \mathcal {W}(V, g)^{\bot }$.

#### Remark 2.13

Let

$$ O(V, g):= \{T \in \operatorname {End}(V): T^*g = g\} $$

be the Lie group of linear isometries of *g*, which acts naturally on curvature tensors. This gives $\mathcal {C}(V)$ the structure of an *O*(*V*, *g*)-module. The curvature decomposition in Proposition 2.12 can be refined to take into account the action of the group *O*(*V*, *g*). It follows that the decomposition in Proposition 2.12 can be extended to

$$ \mathcal {C}(V) = \mathcal {W}(V, g)\oplus \mathcal {W}(V, g)^{\bot } = \mathcal {W}(V, g)\oplus \Sigma ^2_0(V^*) \oplus \mathbb {R}, $$

where the latter is an orthogonal decomposition into irreducible *O*(*V*, *g*)-modules. Note that this implies that the projections onto the submodules are *O*(*V*, *g*)-equivariant.

In this decomposition, the $\Sigma ^2_0(V^*)$-summand represents the trace-free Ricci curvature  of *C* and the $\mathbb {R}$-summand represents the scalar curvature $S_C$ of *C*. For a proof, see [47]; for more context, see [11, Thm. 3.1].

### Pontryagin forms and higher curvature operators on pseudo-Riemannian manifolds

Pontryagin classes are a set of characteristic classes associated with real vector bundles. They are elements of the (de Rham) cohomology of the base space of the vector bundle and are important tools for distinguishing vector bundles both in algebraic topology and differential geometry. From a differential geometric perspective, characteristic classes have the interesting property that they can be computed using the curvature of an arbitrary connection on the vector bundle, but do not depend on the choice of connection. Consequently, the vanishing of some characteristic classes is sometimes a necessary (but certainly not always a sufficient) obstruction for the existence of other geometric structures on that vector bundle. For more context on characteristic classes, we refer to [9, 39, 56].

In our main theorem, Theorem 3.10, we will show that if the Weyl curvature tensor of a pseudo-Riemannian manifold has certain symmetries, then we can ensure the vanishing of specific products of Pontryagin classes. To prove the vanishing of these cohomology classes, we consider a specific differential form representing these cohomology classes and show that these vanish in Theorem 3.9. To make the proof of Theorem 3.9 as clear as possible, the choice of representing differential form is important. Writing the representing differential forms in terms of the higher curvature operators is particularly fruitful for our purposes. Therefore, in this section we will first recall the construction of the Pontryagin classes of a vector bundle in terms of the curvature of a connection and then show how these can be represented using higher curvature operators.

Let *M* be a manifold. Let E→M be a real vector bundle over *M* of rank *r* and let $$\nabla ^E$$ be a connection on *E*. Let $$R^E$$ denote the curvature tensor of $$\nabla ^E$$, which is defined similar to (2.1). Choose any local frame $$e = (e_1, \ldots , e_r)$$ of *E* on some open subset U⊆M. The *curvature matrix* $$\Omega _U$$ of $$R^E$$ on *U* is the matrix of local 2-forms  defined by2.7Clearly, if we choose a different frame $e^{\prime }$ on *U* such that  for some transition matrix , then standard linear algebra shows that the curvature matrix of  on *U* changes by

$$ \Omega '_U = \tau ^{-1}\Omega _U \tau . $$

The Pontryagin classes are defined as the cohomology classes represented by differential forms on *M* that are locally given as a polynomial combination of the local 2-forms . To construct a globally well-defined differential form from these local curvature matrices, that does not depend on the choice of local frame, this polynomial must therefore be invariant under conjugation of the argument. Note that the determinant is such a polynomial. Indeed, the determinant, when viewed as map from $M_r(\mathbb {R})\rightarrow \mathbb {R}$ is conjugation invariant and can be expressed as a polynomial in the matrix entries. Let $t\in \mathbb {R}$ and let $\sigma ^{(r)}_1, \ldots , \sigma ^{(r)}_r:M_r(\mathbb {R})\rightarrow \mathbb {R}$ be the polynomials defined by

$$ \det (I + tX) = 1 + \sum _{k \in [r]}t^k\sigma ^{(r)}_k(X), $$

for all $X \in M_r(\mathbb {R})$. The polynomials $\sigma ^{(r)}_k$ are homogeneous of degree *k* and are invariant under conjugation of *X* with elements of $GL_r(\mathbb {R})$, since the determinant is.

We can now construct the Pontryagin classes as follows. If $\Omega _U \in M_r(\Omega ^2(U))$ is the curvature matrix with respect to an arbitrary local frame *e* on *U*, then the differential form $\sigma ^{(r)}_k(\Omega _U) \in \Omega ^{2k}(U)$ is well-defined since wedge products of differential 2-forms commute, and does not depend on the choice of *e* by conjugation-invariance of $\sigma ^{(r)}_k$. In particular, if *U* and *V* are two trivializing neighborhoods for *E*, then $\sigma ^{(r)}_k(\Omega _U)$ and $\sigma ^{(r)}_k(\Omega _V)$ agree on U∩V and thus the differential forms $\sigma ^{(r)}_k(\Omega _U)$ glue to a global differential form, denoted by $\sigma ^{(r)}_k(\Omega _{\nabla ^E}) \in \Omega ^{2k}(M)$. It can be shown, that the differential form $\sigma ^{(r)}_k(\Omega _{\nabla ^E})$ is a de Rham cocycle [39, p. 296] and that if $\overline{\nabla }^E$ is a second connection on *E*, which induces the differential form $\sigma ^{(r)}_k(\Omega _{\overline{\nabla }^E})$, then the differential form $\sigma ^{(r)}_k(\Omega _{\nabla ^E}) - \sigma ^{(r)}_k(\Omega _{\overline{\nabla }^E})$ is a de Rham coboundary [39, p. 298]. This ensures that the following definition does not depend on the choice of connection.

#### Definition 2.14

Let *M* be an *n*-dimensional manifold and let E→M be a rank *r* vector bundle over *M*. Then for all $1\le k\le \lfloor \frac{r}{2} \rfloor$, the *kth Pontryagin class of E*, denoted by $p_k(E)$, is the class

$$ p_k(E) = \frac{1}{(2\pi )^{2k}}[\sigma ^{(r)}_{2k}(\Omega _{\nabla ^E})] \in H^{4k}_{dR}(M), $$

where $\nabla ^E$ is any connection on *E* and [-] denotes the corresponding de Rham cohomology class. We also define the *k*th Pontryagin class of *M* as

$$ p_k(M):= p_k(TM). $$

For the rest of this section, let (*V*, *g*) be an *n*-dimensional scalar product space and let $C \in \mathcal {C}(V)$ be an algebraic curvature tensor. Motivated by Definition 2.14, we consider the following.

#### Definition 2.15

For all $1\le k\le \lfloor \frac{n}{2} \rfloor$, we define the *k*th *Pontryagin form* of *C* by

$$ \varpi _k(C) = \frac{1}{(2\pi )^{2k}}\sigma ^{(n)}_{2k}(\Omega _C) \in \wedge ^{4k}V^*, $$

where $\Omega _C$ is the curvature matrix of *C*, when viewed as a (1, 3)-tensor, with respect to an arbitrary basis of *V*.

#### Remark 2.16

Note that the $\varpi _k(C)$ not only depends on *C*, but also on the scalar product *g* as it is used to convert *C* to a (1, 3)-tensor.

As mentioned in the beginning of this section, we will show in Theorem 3.9 that certain symmetries of the curvature tensor *C* ensure that products of specific Pontryagin forms of *C* vanish. For the presentation of the argument, it is useful to rewrite the *k*th Pontryagin forms of *C* in terms of the *k*th curvature operator of *C*. This was first done in the Riemannian setting by Stehney [48, Thm. 4.1], based on work by Chern [13, Thm. 2]. The corresponding result in the pseudo-Riemannian case is mentioned by Greub [21, §4] without proof. Therefore, we provide this proof in Appendix A.

Note that the *k*th curvature operator is an endomorphism on $\wedge ^{2k}V$, whereas the *k*th Pontryagin form is a 4*k*-form on *V*. Therefore, consider the following 2*k*-form constructed from two endomorphisms on $\wedge ^k V$.

#### Definition 2.17

Let $A, B \in \operatorname {End}(\wedge ^k V)$. Then $F_k(A, B) \in \wedge ^{2k}V^*$ is the 2*k*-form defined by

2.8 $$\begin{aligned} \begin{array}{c} F_k(A, B)(x_1\wedge \cdots \wedge x_{2k}) \\ = \frac{1}{k!^2}\sum _{\sigma \in S_{2k}}\operatorname {sgn}(\sigma )\langle A(x_{\sigma (1)}\wedge \cdots \wedge x_{\sigma (k)}), B(x_{\sigma (k+1)}\wedge \cdots \wedge x_{\sigma (2k)})\rangle _g \end{array}\end{aligned}$$

for all $$x_1, \ldots , x_{2k} \in V$$.

Using $F_{2k}$, we can express the *k*th Pontryagin form of *C* in terms of its *k*th curvature operator [21, 48].

#### Theorem 2.18

For all $$1\le k\le \lfloor \frac{n}{2} \rfloor $$,

2.9 $$\begin{aligned} \varpi _k(C) = \frac{1}{(2\pi )^{2k}k!^2}F_{2k}(\hat{C}^{*k}, \hat{C}^{*k}). \end{aligned}$$

A proof of Theorem 2.18 is given in Appendix A. We conclude this section by recalling the following result, which shows that the Pontryagin forms of an algebraic curvature tensor $C \in \mathcal {C}(V)$ are equal to the Pontryagin forms of its Weyl curvature tensor $W_C$. This was first shown by Avez [3] in 1970, based on a paper by Chern and Simons [14], and later by Greub [21] and also by Bivens [8, Lem. 2.1.a].

#### Theorem 2.19

Let $C \in \mathcal {C}(V)$ be an algebraic curvature tensor and let $W_C$ be the Weyl curvature tensor of *C* as in Definition 2.9. Then for all $1\le k\le \lfloor \frac{n}{2} \rfloor$,

2.10 $$\begin{aligned} \varpi _k(C) = \varpi _k(W_C). \end{aligned}$$

On manifolds, one can show that Theorem 2.19 implies the following result from Stehney [48, Thm. 4.1] and Greub [21, §4].

#### Corollary 2.20

Let (*M*, *g*) be an *n*-dimensional pseudo-Riemannian manifold and let *Rm* be its Riemann curvature tensor and *W* its Weyl curvature tensor. Then for $1\le k\le \lfloor \frac{n}{2} \rfloor$, the *k*th Pontryagin class of *M* is given by

$$ p_k(M) = \frac{1}{(2\pi )^{2k}k!^2}[F_{2k}(\hat{Rm}^{*k}, \hat{Rm}^{*k})] = \frac{1}{(2\pi )^{2k}k!^2}[F_{2k}(\hat{W}^{*k}, \hat{W}^{*k})]. $$

## Pontryagin forms of even and odd curvatures tensors

In this section, we will first recall the definition of Hervik et al [25] of purely electric and magnetic algebraic curvature tensors. Their approach is to consider the parity of an algebraic curvature tensor under the reflection of a timelike unit vector. In Sect. 3.2, we will show that for a 4*k*-dimensional manifold, the products of Pontryagin classes that land $H_{dR}^{4k}(M)$ vanish if the manifold has a PE or PM Weyl curvature tensor (Theorem 3.10). We actually prove something stronger, namely that under these assumptions, not only these products of Pontryagin classes vanish but in fact the products of the Pontryagin forms as in Definition 2.15 vanish everywhere on the manifold (Theorem 3.9). It is not necessary to restrict ourselves to considering reflections in a timelike unit vector or to manifolds with a Lorentzian signature. Accordingly, in Definition 3.5 we introduce the more general notion of algebraic curvature tensors being even or odd with respect to an endomorphism of the vector space that preserves the scalar product *g* and has determinant -1. Our main results, Theorems 3.9 and 3.10, can then be proved for any such even or odd algebraic curvature tensor. In Sects. 3.3 and 3.4, we will discuss applications of our main results by providing obstructions to the existence of certain Petrov types on 4-dimensional Lorentzian manifolds and nondegenerate umbilic hypersurfaces of pseudo-Riemannian manifolds.

### Motivation: purely electric or magnetic curvature tensors for Lorentzian vector spaces

The orthogonal splitting of the space of curvature tensors $\mathcal {C}(V)$ of a Lorentzian vector space (*V*, *g*) introduced by Hervik et al [25] is based on the reflection in the orthogonal complement of a timelike unit vector u∈V. Concretely, given a timelike unit vector u∈V, we define the reflection $\theta _u$ by $\theta _u(u) = -u$ and ${\theta _u}\Big |_{u^{\bot }} = \operatorname {id}_{u^{\bot }}$. For future reference, we record the following.

#### Lemma 3.1

$$\theta _u \in O(V, g)$$ and $$\det (\theta _u) =-1$$.

Such an endomorphism naturally acts on algebraic curvature tensors via

3.1 $$\begin{aligned} \theta _u^*C(w,x,y,z ) = C( \theta _uw, \theta _ux, \theta _uy, \theta _uz). \end{aligned}$$

It is clear that the (0, 4)-tensor $\theta ^*_uC$ is again an algebraic curvature tensor and that the map $\theta _u^*:\mathcal {C}(V)\rightarrow \mathcal {C}(V)$ is an involution. Therefore, we can decompose *C* as follows.

#### Definition 3.2

i) Let $C \in \mathcal {C}(V)$ be an algebraic curvature tensor and let $C_{\pm } = \frac{C \pm \theta _u^*C}{2}$. Then $C = C_+ + C_-$ is the decomposition of *C* in ±1-eigenvectors of $\theta ^*_u$. We call $C_+$ the *electric part of C with respect to u* and $C_-$ the *magnetic part of C with respect to u*.
ii) We say that *C* is *purely electric (PE) with respect to u* if $C = C_+$ ($C_- = 0$) and that *C* is *purely magnetic (PM) with respect to u* if $C = C_-$ ($C_+ = 0$).
iii) We say that *C* is *PE* or *PM* if there exists a unit timelike vector u∈V such that *C* is PE or PM with respect to *u*.

#### Remark 3.3

i) It is easy to check that an algebraic curvature tensor *C* is PE with respect to *u* if and only if
  C(u,x,y,z)=0
  for all $x, y, z \in u^{\bot }$. Similarly, *C* is PM with respect to *u* if and only if
  C(u,x,y,u)=C(w,x,y,z)=0
  for all $$w, x, y, z \in u^{\bot }$$.
ii) If an algebraic curvature tensor *C* is PE or PM with respect to a unit timelike vector *u*, then so is its associated Weyl curvature tensor $W_C$ [25, Prop. 4.2–3], see also Lemma 3.6.
iii) For Weyl curvature tensors on a 4-dimensional Lorentzian vector space, the conventional definition of the electric and magnetic parts, see Matte [37, §4], is equivalent to Definition 3.2 [25, Rem. 3.2].

#### Example 3.4

Let $I\times _f \Sigma$ be the Lorentzian warped product with an open interval $I\subset \mathbb {R}$ as base and an arbitrary Riemannian manifold $(\Sigma , \sigma )$ as fiber with the metric $g = -dt^2 + f(t)^2\sigma$. Then, $I\times _f \Sigma$ has a PE Weyl curvature tensor with respect to the unit timelike vector field $\partial _t$.

More generally, every spacetime *M* that admits a unit timelike congruence $u \in \mathfrak {X}(M)$ that is shear-free and irrotational has a Weyl curvature tensor that is PE with respect to *u* [25, Prop. 3.17]. Other examples of spacetimes with a PE or PM Weyl curvature tensor are spacetimes with irrotational perfect fluids [4, 5, 31, 32].

### A vanishing theorem for Pontryagin forms of even/odd curvature tensors

In this section, we prove in Theorem 3.9 that if an algebraic curvature tensor *C* is θ-even or -odd (in the sense of Definition 3.5), θ has determinant -1, and the dimension of the vector space is a multiple of 4, then any product of Pontryagin forms of *C* that lands in the top exterior power of the dual vector space vanishes. This can be proved generally for all algebraic curvature tensors. As a consequence, we derive in Theorem 3.10 the vanishing of products of Pontryagin classes on compact and orientable pseudo-Riemannian manifolds that have PE or PM Weyl curvature tensors. In Sects. 3.3 and 3.4, these results are related to the Petrov classification for 4-dimensional Lorentzian manifolds and nondegenerate umbilic hypersurfaces of pseudo-Riemannian manifolds.

For the rest of this section, let (*V*, *g*) be a scalar product space of dimension *n*.

#### Definition 3.5

Let $C \in \mathcal {C}(V)$ be an algebraic curvature tensor and let $\theta \in O(V, g)$. We say that *C* is θ*-even* if

$$ \theta ^*C(u,x,y,z) = C(u,x,y,z), $$

for all u,x,y,z∈V. Similarly, we say that *C* is θ*-odd* if

$$ \theta ^*C(u,x,y,z) = -C(u,x,y,z), $$

for all u,x,y,z∈V.

By Lemma 3.1, we see that a PE or PM algebraic curvature tensor $C \in \mathcal {C}(V)$ is θ-even or -odd with respect to an orientation-reversing isometry θ of *V*, respectively.

Like being PE or PM, being θ-even- or -odd also descends to Weyl curvature tensors (see part ii) of Remark 3.3).

#### Lemma 3.6

Let $C \in \mathcal {C}(V)$ be an algebraic curvature tensor and let $\theta \in O(V, g)$. If *C* is θ-even or -odd, then so is its Weyl curvature tensor $W_C$.

#### Proof

The map $\mathcal {W}:\mathcal {C}(V)\rightarrow \mathcal {W}(V, g)$ mapping an algebraic curvature tensor *C* to its Weyl curvature tensor $W_C$ is a linear map. Indeed, by linearity of the trace, both the Ricci curvature ${{\,\textrm{Ric}\,}}_C$ and scalar curvature $S_C$ depend linearly on *C*. Therefore, also the Schouten tensor

$$ P_C = \frac{1}{n-2}\left( {{\,\textrm{Ric}\,}}_C-\frac{S_C}{2(n-1)}g\right) $$

depends linearly on *C*. Finally, by bilinearity of the Kulkarni–Nomizu product, it follows thatdepends linearly on *C*. Moreover, the decomposition theorem from Remark 2.13 ensures that the map $\mathcal {W}:\mathcal {C}(V)\rightarrow \mathcal {W}(V, g)$ is *O*(*V*, *g*)-equivariant. It follows that if *C* is θ-even or -odd, then

$$ \theta ^*W_C = \theta ^*\mathcal {W}(C) = \mathcal {W}(\theta ^*C) = \pm \mathcal {W}(C) = \pm W_C.$$

□

The following result is an easy consequence of the definitions.

#### Lemma 3.7

Let $C \in \mathcal {C}(V)$ be an algebraic curvature tensor and let $\hat{C}$ be the induced curvature operator. Let $\theta \in O(V, g)$. If *C* is θ-even or -odd, then

3.2 $$\begin{aligned} \hat{C}\circ \wedge ^2\theta = \pm \wedge ^2\theta \circ \hat{C}, \end{aligned}$$

where the positive sign corresponds to the θ-even case and the negative sign to the θ-odd case.

#### Lemma 3.8

Let $C \in \mathcal {C}(V)$ be an algebraic curvature tensor and let $\theta \in O(V,g)$. If *C* is θ-even or -odd, then for all $1\le k\le \lfloor \frac{n}{2} \rfloor$ we have

$$ \wedge ^{4k}\theta ^*(\varpi _k(C)) = \varpi _k(C). $$

#### Proof

An easy computation using Theorem 2.18 shows that

$$\begin{aligned} \wedge ^{4k}\theta ^*(\varpi _k(C))&= \frac{1}{(2\pi )^{2k}k!^2}\wedge ^{4k}\theta ^*(F_{2k}(\hat{C}^{*k}, \hat{C}^{*k}))\\&\overset{{\text {Def. 2.17}}}{=} \frac{1}{(2\pi )^{2k}k!^2}F_{2k}(\hat{C}^{*k}\circ \wedge ^{2k}\theta , \hat{C}^{*k}\circ \wedge ^{2k}\theta )\\&\overset{(2.5)}{=}\ \frac{1}{(2\pi )^{2k}k!^2}F_{2k}((\hat{C}\circ \wedge ^{2}\theta )^{*k}, (\hat{C}\circ \wedge ^{2}\theta )^{*k})\\&\overset{(3.2)}{=}\ \frac{1}{(2\pi )^{2k}k!^2}F_{2k}((\pm 1)^k(\wedge ^{2}\theta \circ \hat{C})^{*k}, (\pm 1)^k(\wedge ^{2}\theta \circ \hat{C})^{*k})\\&\overset{(2.6)}{=}\ \frac{(\pm 1)^{2k}}{(2\pi )^{2k}k!^2}F_{2k}(\wedge ^{2k}\theta \circ \hat{C}^{*k}, \wedge ^{2k}\theta \circ \hat{C}^{*k})\\&=\frac{1}{(2\pi )^{2k}k!^2}F_{2k}(\hat{C}^{*k}, \hat{C}^{*k}) = \varpi _k(C), \end{aligned}$$

where the first equality on the last line follows from the fact that $\wedge ^{2k}\theta$ is an isometry for $\langle -,-\rangle _g$ as θ is an isometry for *g*. □

For a multi-index of positive integers $\alpha = (\alpha _1, \ldots , \alpha _l) \in \mathbb {Z}_{>0}^l$ of length *l*, we denote its *absolute value* by

3.3 $$\begin{aligned} |\alpha | = \sum _{i = 1}^l\alpha _i. \end{aligned}$$

Note that if |α|=k, then the length of α is less than or equal to *k* and none of the entries of α are greater than *k*.

In Lemma 3.8, we showed that if *C* is a θ-even or -odd algebraic curvature tensor, then the Pontryagin forms $\varpi _k(C)$ of *C* are fixed by the map $\wedge ^{4k}\theta ^*$ induced on $\wedge ^{4k}V^*$ by θ. This also means that products of Pontryagin forms that land in $\wedge ^{4l}V^*$ for some integer *l* are fixed by the map $\wedge ^{4l}\theta ^*$ induced on $\wedge ^{4l}V^*$ by θ. If *V* is *n*-dimensional, then $\wedge ^nV^*$ is 1-dimensional and the induced map $\wedge ^n\theta ^*$ is nothing but multiplication by $\det (\theta ^*) = \det (\theta )$ [22, Ch. 7.2]. Finally, if $\theta \in O(V,g)$, we have either $\det (\theta ) = \pm 1$. So if $\det (\theta ) = -1$, we obtain the following result on the vanishing of (products of) Pontryagin forms.

#### Theorem 3.9

(Vanishing theorem Pontryagin forms). Let *V* be 4*k*-dimensional and let $\theta \in O(V,g)$ with $\det (\theta ) = -1$. Suppose that *C* is an algebraic curvature tensor that is θ-even or -odd. Then for all multi-indices α with |α|=k:

3.4 $$\begin{aligned} \varpi _{\alpha }(C):= \varpi _{\alpha _1}(C)\wedge \cdots \wedge \varpi _{\alpha _l}(C) = 0 \in \wedge ^{4k}V^*. \end{aligned}$$

#### Proof

Since $\varpi _{\alpha }(C) \in \wedge ^{4k}V^*$, which is 1-dimensional as *V* is 4*k*-dimensional. It follows that $\wedge ^{4k}\theta ^*$ is nothing but multiplication with $\det (\theta ^*) = -1$. So on the one hand, we have

3.5 $$\begin{aligned} \wedge ^{4k}\theta ^*(\varpi _{\alpha }(C)) = \det (\theta ^*)\varpi _{\alpha }(C) = -\varpi _{\alpha }(C). \end{aligned}$$

On the other hand, by Lemma 3.8,

3.6 $$\begin{aligned} \wedge ^{4k}\theta ^*(\varpi _{\alpha }(C)) = \wedge ^{4\alpha _1}\theta ^*(\varpi _{\alpha _1}(C))\wedge \cdots \wedge (\wedge ^{4\alpha _l}\theta ^*(\varpi _{\alpha _l}(C))) = \varpi _{\alpha }(C). \end{aligned}$$

The result follows by combining (3.5) and (3.6). □

Now, if (*M*, *g*) is a 4*k*-dimensional pseudo-Riemannian manifold and if there is some $\theta \in \operatorname {End}(TM)$ satisfying the conditions of Theorem 3.9 at each p∈M for which the Weyl curvature tensor of *M* is even or odd. Then by applying Theorem 3.9 to the tangent spaces of *M*, we obtain the following result on the vanishing of products of Pontryagin classes.

#### Theorem 3.10

(Vanishing theorem Pontryagin classes). Let (*M*, *g*) be a 4*k*-dimensional pseudo-Riemannian manifold and let $\theta \in \operatorname {End}(TM)$ with $\theta _p \in O(T_pM, g_p)$ and $\det (\theta _p) = -1$ for all p∈M. Suppose that the Weyl curvature tensor *W* is θ-even or -odd at each p∈M. Then for all multi-indices α with |α|=k,

3.7 $$\begin{aligned} \varpi _{\alpha }(W) = 0 \in \Omega ^{4k}(M). \end{aligned}$$

In particular, the product of Pontryagin classes

3.8 $$\begin{aligned} p_{\alpha }(M):= p_{\alpha _1}(M)\smile \cdots \smile p_{\alpha _l}(M) = 0 \in H^{4k}_{dR}(M). \end{aligned}$$

#### Proof

The identity (3.7) follows directly by applying Theorem 3.9 to $(T_pM, g_p)$ for each p∈M. Using Corollary 2.20 and (3.7), we see that

$$ p_{\alpha _1}(M)\smile \cdots \smile p_{\alpha _l}(M) = [\varpi _{\alpha _1}(W)\wedge \cdots \wedge \varpi _{\alpha _l}(W)] = [0] = 0. $$

□

#### Remark 3.11

Let us compare how Theorem 3.10 relates to existing results in the literature.

i) Mantica and Molinari introduced the notion of a *C*-*compatible vector* as a special case of the more general notion of *C*-*compatible symmetric (0, 2)-tensors* which are studied in [35, 36]. If u∈V is not null and *C*-compatible, then
  C(u,x,y,z)=0
  for all $x, y, z \in u^{\bot }$ [36, Thm. 3.4]. An easy computation using the algebraic Bianchi identity for algebraic curvature tensors shows that the converse also holds. So for timelike unit vectors u∈V, *C*-compatibility is equivalent to *C* being PE with respect to *u* by part i) of Remark 3.3. They also derive a theorem relating the existence of *C*-compatible vectors to the vanishing of Pontryagin classes [36, Thm. 3.6]. Their theorem differs from our Theorem 3.10 in that they use the very strong assumption of having, around each point p∈M, a local orthonormal frame where all but two frame fields are *Rm*-compatible. Using this assumption, they conclude that the Riemann tensor is *pure*—that is, the Riemann curvature operator $\hat{Rm}$ is diagonal with respect to an orthonormal basis of $\wedge ^2T_pM$ induced by an orthonormal basis of $T_pM$. A theorem of Maillot [34] then ensures the vanishing of all Pontryagin forms and classes.
ii) If the manifold *M* in Theorem 3.10 is 4-dimensional, then the conclusion is that $p_1(M)$ vanishes. For 4-dimensional Lorentzian manifolds *M*, it was already known that the Pontryagin class $p_1(M)$ vanishes if the manifold admits a Lorentzian metric with a PE or PM Weyl curvature tensor. This follows directly from [57, p. 323], but is not mentioned explicitly. Our Theorem 3.10 extends this result to higher-dimensional manifolds, all pseudo-Riemannian signatures and to a broader class of symmetries.

#### Remark 3.12

(Theorem 3.10 for the Riemann tensor *Rm*). If (*M*, *g*) and θ are as in Theorem 3.10 but now the Riemann curvature tensor *Rm* is θ-even or -odd at each point p∈M, then we can also apply Theorem 3.9 pointwise to *Rm* to draw the same conclusion as in Theorem 3.10. Alternatively, in this case Theorem 3.10 can also be applied directly as the Weyl curvature tensor *W* is also θ-even or -odd by Lemma 3.6.

In the light of Mantica and Molinari’s vanishing result on Pontryagin classes [36, Thm. 3.6] discussed in part i) of Remark 3.11, it is natural to ask whether or not our Theorem 3.10 can be sharpened. Example 3.13 shows that the conclusion of Theorem 3.10 is optimal in the sense that under the stated assumptions, all products of Pontryagin classes of degree strictly less than 4*k* need not vanish. However, it remains open whether the assumptions on *W* can be weakened.

#### Example 3.13

(Theorem 3.10 is optimal). If k=1 in Theorem 3.10, then there are no Pontryagin forms of degree less then 4*k*. So to give an example of a pseudo-Riemannian manifold of dimension 4*k* satisfying the assumptions of Theorem 3.10 for which all products of Pontryagin forms of degree less than 4*k* do not vanish, we need at least k≥2. We give an example for any k≥2.

Let k≥2 be a positive integer. We construct a Lorentzian manifold (*M*, *g*) of dimension 4*k* that has a PE Weyl curvature tensor and such that for all multi-indices α with |α|<k, the class

$$ p_\alpha (M) \ne 0 \in H^{4|\alpha |}_{dR}(M). $$

Then in particular, for all multi-indices α with |α|<k, the 4|α|-form

$$ \varpi _\alpha (M) \ne 0 \in \Omega ^{4|\alpha |}(M). $$

First, consider the manifold $\mathbb {C}P^{2k-2}$, which is of real dimension 4k-4. It can be shown [39, p. 185] that for all multi-indices α with $|\alpha | \le k-1$

$$ p_{\alpha }(\mathbb {C}P^{2k-2})\ne 0. $$

Equip $\mathbb {C}P^{2k-2}$ with an arbitrary Riemannian metric *h* and use the 4-dimensional Minkowski space $(\mathbb {R}^{1, 3}, \eta )$ to form the 4*k*-dimensional Lorentzian product manifold

$$ (M, g) = (\mathbb {R}^{1, 3}\times \mathbb {C}P^{2k-2}, \eta \oplus h), $$

which has a PE Weyl curvature tensor by Example 3.4.

We will show that for all multi-indices α with $|\alpha | \le k-1$

$$ p_{\alpha }(M)\ne 0 \in H^{4|\alpha |}_{dR}(M). $$

Denote by *p*(*M*), $p(\mathbb {R}^{1, 3})$ and $p(\mathbb {C}P^{2k-2})$ the total Pontryagin classes of these manifolds and denote by $\pi _1 :M\rightarrow \mathbb {R}^{1, 3}$ and $\pi _2:M\rightarrow \mathbb {C}P^{2k-2}$ the projection maps. Using standard properties of Pontryagin classes (see, for example, [39, §15]), we see that

3.9 $$\begin{aligned} p(M)&= p(\pi _1^*T\mathbb {R}^{1,3}\oplus \pi ^*_2T\mathbb {C}P^{2k-2}) \nonumber \\&= \pi _1^*p(\mathbb {R}^{1,3})\smile \pi ^*_2p(\mathbb {C}P^{2k-2}) = \pi ^*_2p(\mathbb {C}P^{2k-2}), \end{aligned}$$

where the last equality follows as $p(\mathbb {R}^{1, 3}) = 1$ as $\mathbb {R}^{1, 3}$ has a trivial tangent bundle. Since the projection $\pi _2$ is a homotopy equivalence, the induced map $\pi _2^*:H^{\bullet }_{dR}(\mathbb {C}P^{2k-2}) \xrightarrow {\sim } H^{\bullet }_{dR}(M)$ is an algebra isomorphism. It follows that for every multi-index α with $|\alpha |\le k-1$,

$$ p_\alpha (M) = \pi _2^*(p_\alpha (\mathbb {C}P^{2k-2})) \ne 0, $$

as $p_\alpha (\mathbb {C}P^{2k-2}) \ne 0$ [39, p. 185] and $\pi _2^*$ is injective. We conclude that for all multi-indices α with |α|<k, the form

$$ \varpi _\alpha (M) \ne 0 \in \Omega ^{4|\alpha |}(M). $$

As a corollary to Theorem 3.10, we find a new obstruction result for PE or PM metrics for compact orientable manifolds.

#### Corollary 3.14

(Pontryagin class obstruction for PE/PM metrics). Let *M* be a compact and orientable 4*k*-dimensional manifold. Suppose that there exists a multi-index α with |α|=k such that

3.10 $$\begin{aligned} p_{\alpha }(M) \ne 0 \in H^{4k}_{dR}(M). \end{aligned}$$

Then *M* does not admit a pseudo-Riemannian metric such that the Weyl curvature tensor is *PE* or *PM* at all points p∈M.

#### Remark 3.15

We present some comments on Corollary 3.14.

i) Corollary 3.14 is only nontrivial for compact orientable manifolds *M*. If an *n*-dimensional manifold *M* is either noncompact or nonorientable, then always $H^{n}_{dR}(M) = 0$ (see for example [23, Prop. 5.IX]).
ii) The obstruction in the Pontryagin classes presented in Corollary 3.14 does not distinguish between PE or PM Weyl curvature tensors. It would be interesting to find obstructions that can distinguish between the (non)existence of PE and PM Weyl curvature tensors separately.

We conclude this section by constructing an example of a 4-dimensional compact orientable manifold that admits Lorentzian metrics, but no such has a PE or PM Weyl curvature tensor. Recall that a compact manifold *M* admits a Lorentzian metric if and only if its Euler characteristic, χ(M), vanishes (see, e.g., [40, Prop. 5.37]). In dimension 4, our goal therefore amounts to constructing a manifold *M* such that

$$ \chi (M) = 0 \quad {\text {and}}\quad p_1(M) \ne 0. $$

To determine the nonvanishing of $p_1(M)$, we need a few tools from differential topology. Recall that for a compact orientable 4-dimensional manifold, integration over *M* determines a linear isomorphism $\int _M:H^4_{dR}(M)\rightarrow \mathbb {R}$ and that under this isomorphism, the cup product on $H^2_{dR}(M)$ can be identified with a symmetric bilinear form *B* on $H^2_{dR}(M)$, which is nondegenerate by Poincaré duality for de Rham cohomology [23, Ch. 5, §4, 5]. It follows that the matrix of *B* is diagonalizable with nonzero eigenvalues. Denote by $n_+$ the number of positive eigenvalues of *B* and by $n_-$ the number of negative eigenvalues of *B*. Then, the *topological signature* of *M* is defined as the integer $\sigma (M) = n_+ - n_-$. In dimension 4, the Hirzebruch signature theorem [26, Thm. 8.2.2] then states that

3.11 $$\begin{aligned} \sigma (M) = \frac{1}{3}\int _Mp_1(M), \end{aligned}$$

and therefore $\sigma (M)\ne 0$ if and only if $p_1(M)\ne 0$ as $\int _M$ is an isomorphism.

Finally, if $M_1$ and $M_2$ are 4-dimensional manifolds, denote their connected sum by $M_1 \# M_2$. It can be shown that

3.12 $$\begin{aligned} \chi (M_1 \# M_2)&= \chi (M_1) + \chi (M_2) - 2,\end{aligned}$$

3.13 $$\begin{aligned} \sigma (M_1 \# M_2)&= \sigma (M_1) + \sigma (M_2), \end{aligned}$$

see for example [24, Exc. 3.3.6] and [27, Thm. 5.3], respectively.

#### Example 3.16

*(A compact manifold without PE or PM Lorentzian metrics).* Consider the 4-dimensional manifolds $\mathbb {T}^4$ and $\mathbb {C}P^2$. It can be shown that

3.14 $$\begin{aligned} \chi (\mathbb {T}^4) = 0&\qquad \chi (\mathbb {C}P^2) = 3\end{aligned}$$

3.15 $$\begin{aligned} \sigma (\mathbb {T}^4) = 0&\qquad \sigma (\mathbb {C}P^2) = 1, \end{aligned}$$

where the Euler characteristics follow from the well-known cell-structures of $\mathbb {T}^4$ and $\mathbb {C}P^2$; $\sigma (\mathbb {T}^4) = 0$ follows from the fact that $\mathbb {T}^4$ has a trivial tangent bundle, hence $p_1(\mathbb {T}^4) = 0$; and for $\sigma (\mathbb {C}P^2)$, see [39, p. 185] and use the Hirzebruch signature theorem. Consider the 4-dimensional manifold $M =\mathbb {T}^4 \#\mathbb {T}^4 \# \mathbb {C}P^2 \# \mathbb {C}P^2$. It follows from (3.12) and (3.14) that χ(M)=0 and from (3.13) and (3.15) that σ(M)=2. So *M* admits Lorentzian metrics, but none that has an everywhere PE or PM Weyl curvature tensor.

### Obstructions to the existence of Petrov types of 4-dimensional Lorentzian manifolds

Corollary 3.14 formulated an obstruction for the existence of metrics that globally have a PE or PM Weyl curvature tensor in terms of the nonvanishing of products of Pontryagin classes. To conclude this section, we will discuss two applications by proving related obstruction results for certain Petrov types for 4-dimensional Lorentzian manifolds and for foliations by nondegenerate umbilic hypersurfaces.

The Petrov classification, introduced by Petrov [43, Ch. 3], is an algebraic classification of Weyl curvature tensors of 4-dimensional Lorentzian manifolds into several types. There are different equivalent approaches to the Petrov classification, also by Bel [7], Debever [17], Penrose [42], Pirani [44] and others (see Batista [6, Ch. 2] for a survey of 6 different approaches). We follow the approach by Thorpe [51], which makes use of the curvature operator introduced in Definition 2.2.

Let (*V*, *g*) be a 4-dimensional Lorentzian vector space. Choose an orientation-defining unit vector $\omega \in \wedge ^4V$. Let $\star \in \operatorname {End}(\wedge ^2V)$ denote the Hodge-star, which is uniquely defined [41, Prop. 5.2.2] by

$$ \xi \wedge \star \eta = -\langle \xi , \eta \rangle _g\omega $$

for all $\xi , \eta \in \wedge ^2V$. It is easy to show that ⋆ is self-adjoint and $\star ^2 = -1$ [41, Lem. 5.2.3]. This means we can view $\wedge ^2V$ as a complex vector space of dimension 3 by declaring i:=⋆. This construction is special for 4-dimensional Lorentzian vector spaces. It is crucial that *V* is 4-dimensional for ⋆ to be an endomorphism of $\wedge ^2V$ and that *V* is Lorentzian for ⋆ to square to -1.

Let $W \in \mathcal {W}(V, g)$ be a Weyl curvature tensor. That *W* is trace-free with respect to *g* implies that $[\hat{W}, \star ] =0$ [41, Cor. 5.3.3] and, therefore, the curvature operator $\hat{W}$ is $\mathbb {C}$-linear. Moreover, by combining the trace-freeness of *W* with the first Bianchi identity, it follows that $\hat{W}$ is trace-free as $\mathbb {C}$-linear operator [41, Rem. 5.4.1]. The Petrov type of *W* is then determined by the Jordan normal form of the curvature operator $\hat{W}$ on $\wedge ^2V$. We distinguish the following different Petrov types [51]:

i) Type *O*: $$\hat{W} = 0$$;
ii) Type *I*: $\hat{W}$ is diagonalizable with 3 different complex eigenvalues;
iii) Type *D*: $\hat{W}$ is diagonalizable with 2 different complex eigenvalues;
iv) Type *II*: $\hat{W}$ is has 2 Jordan blocks with 2 different complex eigenvalues;
v) Type *N*: $\hat{W}$ is has 2 Jordan blocks with the same complex eigenvalue (necessarily 0); and
vi) Type *III*: $\hat{W}$ is has 1 Jordan block (necessarily with 0 as eigenvalue).

Note that this list is exhaustive as there are at most 3 Jordan blocks, in which case $\hat{W}$ is diagonalizable, and if $\hat{W}$ has one complex eigenvalue with multiplicity 3, then this eigenvalue is necessarily 0 as $\hat{W}$ is trace-free as $\mathbb {C}$-linear operator.

Many of the Petrov types imply the vanishing of the Pontryagin form $\varpi _1(W)$. Our results in Sect. 3.2 now allow us to include certain subtypes of type *I* to this list.

#### Theorem 3.17

Let *W* be a Weyl curvature tensor on a 4-dimensional Lorentzian vector space (*V*, *g*). Then $\varpi _1(W) = 0$, if *W* has either of the following Petrov types:

i) Type *O*,
ii) Type *I* or *D* with real or imaginary eigenvalues,
iii) Type *II* with real or imaginary eigenvalues,
iv) Type *N*,
v) Type *III*.

#### Proof

For type *O*, *N*, *III* and type *D* and *II* with real or imaginary eigenvalues, this was proved by Avez [3, Cor. 4], Zund [58, Thm. 2] and Porter and Thompson [45, Thm. 3–5].

If *W* has type *I* with real or imaginary eigenvalues, then it follows that *W* is PE or PM, respectively, see for example [55, 25, Rem. 3.8] or [38, p. 1556]. So $\varpi _1(W)=0$ by Theorem 3.9. □

#### Corollary 3.18

(Pontryagin class obstruction for Petrov types). Let *W* be the Weyl curvature tensor of a 4-dimensional Lorentzian manifold (*M*, *g*). Then $p_1(M) = 0$, if *W* has either of the following Petrov types globally:

i) Type *O*,
ii) Type *I* or *D* with real or imaginary eigenvalues,
iii) Type *II* with real or imaginary eigenvalues,
iv) Type *N*,
v) Type *III*.

Conversely, if *M* is a compact orientable 4-dimensional manifold such that $p_1(M)\ne 0$, then *M* does not admit Lorentzian metrics that are globally of the Petrov types listed above. □

For example, the manifold constructed in Example 3.16 does not admit Lorentzian metrics with a Weyl curvature tensor that has any of the Petrov types listed in Corollary 3.18 globally.

### Obstructions to the existence of foliations by nondegenerate umbilic hypersurfaces of pseudo-Riemannian manifolds

Let (*M*, *g*) be a pseudo-Riemannian manifold and consider a hypersurface $\Sigma \hookrightarrow M$. Denote $\sigma = {g}\Big |_{\Sigma }$ and assume that σ is a pseudo-Riemannian metric. Then, the tangent bundle of *M* over Σ splits as

3.16 $$\begin{aligned} {TM}\Big |_{\Sigma } = T\Sigma \oplus \mathcal {N}\Sigma , \end{aligned}$$

where $\mathcal {N}\Sigma$ denotes the normal bundle of Σ in *M*. Using the splitting (3.16), we can decompose the Levi-Civita connection of *M* into its tangential and normal components at Σ. More precisely, let $X, Y\in \mathfrak {X}(\Sigma )$ and extend *X* and *Y* arbitrarily to vector fields *X* and *Y* of an open neighborhood of Σ in *M*. Then

3.17 $$\begin{aligned} {\nabla ^M_XY}\Big |_{\Sigma } = \nabla ^\Sigma _XY + \operatorname {II}(X, Y), \end{aligned}$$

where the first term on the right-hand side of (3.17) is the Levi-Civita connection of $(\Sigma , \sigma )$ and the second term is the *second fundamental form of the embedding*. As $\mathcal {N}\Sigma$ is a line bundle, we can locally fix a unit normal vector *N* to Σ generating $\mathcal {N}\Sigma$, and therefore, the second fundamental form takes the shape

3.18 $$\begin{aligned} \operatorname {II}(X, Y) = h(X, Y)N, \end{aligned}$$

for all $X,Y \in \mathfrak {X}(\Sigma )$, where *h* is a symmetric (0, 2)-tensor field on Σ [29, Prop. 8.1].

#### Definition 3.19

A nondegenerate hypersurface $\Sigma \hookrightarrow M$ is *umbilic* if around each point $p\in \Sigma$ there is a neighborhood $U\subseteq \Sigma$ with a fixed unit normal vector field *N* such that the scalar second fundamental form *h* is of the form h=fσ for some smooth function $f \in C^{\infty }(U)$. If on any such neighborhood f≡0, then Σ is *totally geodesic*.

Umbilic hypersurfaces are studied both in the setting of Riemannian geometry ([12, 20, 28] are just a few examples) and in the setting of Lorentzian geometry, where umbilic spacelike hypersurfaces may appear as time slices of spacetimes [2, 10, 19]. On an umbilic nondegenerate hypersurface, the Weyl curvature tensor was shown in [46, §2] to satisfy

3.19 $$\begin{aligned} W(N, X, Y, Z) = 0 \end{aligned}$$

for all $X, Y, Z \in \mathfrak {X}(\Sigma )$. The proof given in [46] is a long and direct computation. There is a more elegant way to see this, which we present in the following theorem. Note that by similar reasoning as in part i) of Remark 3.3, (3.19) is equivalent to *W* being even under $\theta \in \operatorname {End}({TM}\Big |_{\Sigma })$ defined by θ(N)=-N and ${\theta }\Big |_{T\Sigma } = \operatorname {id}_{T\Sigma }$, which is a globally well-defined vector bundle morphism by (3.16) even though *N* is defined only locally.

#### Theorem 3.20

Suppose that $\Sigma \hookrightarrow M$ is a nondegenerate umbilic hypersurface. Then, the Weyl curvature tensor *W* of *M* is θ-even on Σ.

#### Proof

Let $p \in \Sigma$ be an arbitrary point. The question of whether or not *W* is θ-even at *p* depends only pointwise on *W*. Therefore, without loss of generality, we may assume (after possibly passing to a neighborhood of *p*) that a unit normal vector field *N* of Σ exists globally on Σ and therefore $\mathcal {N}\Sigma \cong \Sigma \times \mathbb {R}$.

By the discussion above, we need to show that

W(N,X,Y,Z)=0

for all $X, Y, Z \in \mathfrak {X}(\Sigma )$ and for the fixed unit normal vector field *N*. Recall that the Codazzi equation reads

3.20 $$\begin{aligned} Rm(X, Y, Z, N) = g(N, N)(Dh)(Z, X, Y), \end{aligned}$$

where $(Dh)(Z, X, Y) = -(\nabla h)(Z, X, Y) + (\nabla h)(Z, Y, X)$ is the *exterior covariant derivative* of *h* [29, p. 236]. Since Σ is an umbilic hypersurface in *M* with a global unit normal vector field *N*, there exists a smooth function $\phi \in C^{\infty }(M)$ such that Σ is totally geodesic with respect to the conformally equivalent metric $\tilde{g} = e^{2\phi }g$ and $\tilde{h}\equiv 0$, where $\tilde{h}$ is the second fundamental form of Σ with respect to $\tilde{g}$. This is shown, for example, in [15] using tractor calculus, but a more elementary proof of this statement is given in Lemma 3.21 after the proof of this theorem.

Let $\tilde{N} = e^{-\phi }N$ be the rescaled unit normal vector field to Σ with respect to $\tilde{g}$ and let $\widetilde{Rm}$ and $\widetilde{W}$ denote the Riemann and Weyl curvature tensors of $\tilde{g}$. By (3.20), we obtain

$$ \widetilde{Rm}(X, Y, Z, \tilde{N}) = \tilde{g}(\tilde{N}, \tilde{N})(D\tilde{h})(Z, X, Y) = 0. $$

As $\tilde{N}$ and *N* are scalar multiples of one another, it follows that $\widetilde{Rm}$ is θ-even on Σ by similar reasoning as in part i) of Remark 3.3. Hence, Lemma 3.6 implies that $\widetilde{W}$ is also θ-even on Σ. Since the Weyl curvature tensor is conformally invariant, we conclude that *W* is θ-even on Σ. □

#### Lemma 3.21

Suppose that $\Sigma \hookrightarrow M$ is a nondegenerate umbilic hypersurface with a global unit normal vector field *N*. Then, there exists a smooth function $\phi \in C^{\infty }(M)$ such that Σ is totally geodesic with respect to the conformally equivalent metric $\tilde{g} = e^{2\phi }g$.

#### Proof

Let $\phi \in C^\infty (M)$ be an arbitrary function and $\tilde{g} = e^{2\phi }g$ the corresponding conformally equivalent metric. Let $\tilde{N} = e^{-\phi }N$ be the rescaled unit normal vector field to Σ with respect to $\tilde{g}$. We will first relate the scalar second fundamental forms *h* of *g* and $\tilde{h}$ of $\tilde{g}$. Note that the Levi-Civita connection ∇ of the metric *g* and the Levi-Civita connection $\widetilde{\nabla }$ of the metric $\tilde{g}$ are related by [29, Prop. 7.29]

3.21 $$\begin{aligned} \widetilde{\nabla }_XY = \nabla _XY + Y(\phi )X+X(\phi )Y-g(X, Y)\nabla \phi \end{aligned}$$

for all $X, Y \in \mathfrak {X}(M)$. By using (3.17) and (3.18) twice on (3.21) and comparing normal components, we find that the scalar second fundamental forms of Σ with respect to the metrics $\tilde{g}$ and *g* are related by

3.22 $$\begin{aligned} e^{-\phi }\tilde{h}(X, Y) = h(X, Y)-g(N, N)g(\nabla \phi , N)\sigma (X, Y) \end{aligned}$$

for all $X, Y \in \mathfrak {X}(\Sigma )$. Since Σ is umbilic with respect *g*, it follows that there is a smooth function $f \in C^{\infty }(\Sigma )$ such that h=fσ. Substituting into (3.22), we find that

3.23 $$\begin{aligned} e^{-\phi }\tilde{h}(X, Y) = \left( f-g(N,N)g(\nabla \phi , N)\right) \sigma (X, Y). \end{aligned}$$

From (3.23), it follows that it is left to show that for all $f \in C^\infty (\Sigma )$, we can find a $\phi \in C^\infty (M)$ such that

3.24 $$\begin{aligned} g(\nabla \phi , N) = g(N,N)f \end{aligned}$$

on Σ.

Indeed, let U⊆M be a tubular neighborhood of Σ with domain $\Sigma \times \{0\} \subset \Sigma _0 \subset \Sigma \times \mathbb {R}$ such that the exponential map

$$ E:\Sigma _0\rightarrow U\qquad (p, t)\mapsto \exp _p(tN) $$

is a diffeomorphism. Note that using this tubular neighborhood, $N = {\partial _t}\Big |_{\Sigma }$. Consider the function $\phi _0:U\rightarrow \mathbb {R}$ defined by

$$ \phi _0(E(p, t)) = g(N,N)f(p)t. $$

We see that

$$\begin{aligned} g(\nabla \phi _0, N) = {g(\nabla \phi _0, \partial _t)}\Big |_{\Sigma } = {\partial _t(\phi _0)}\Big |_{\Sigma } = g(N, N)f \end{aligned}$$

and therefore $\phi _0$ satisfies (3.24). So we obtain ϕ by extending $\phi _0$ to a function on all of *M* by using a partition of unity that does not change $\phi _0$ in a neighborhood of Σ. □

Combining Theorems 3.9 and 3.20, we find that for all multi-indices α with |α|=k, the 4*k*-form $\varpi _{\alpha }(W)$ vanishes at a nondegenerate umbilic hypersurface of a 4*k*-dimensional pseudo-Riemannian manifold.

#### Theorem 3.22

(Pontryagin forms vanish at nondegenerate umbilic hypersurfaces). Let (*M*, *g*) be a 4*k*-dimensional pseudo-Riemannian manifold. Let $\Sigma \hookrightarrow M$ be a nondegenerate umbilic hypersurface. Then for all multi-indices α with |α|=k, the 4*k*-form $\varpi _{\alpha }(W)$ vanishes at $\Sigma \subseteq M$. □

Now suppose that a pseudo-Riemannian manifold (*M*, *g*) is foliated by nondegenerate umbilic hypersurfaces. Then, every point of *M* lies in a unique leaf of the foliation, which is a nondegenerate umbilic hypersurface. So Theorem 3.22 yields that for all multi-indices α with |α|=k, the 4*k*-form $\varpi _{\alpha }(W)$ vanishes everywhere on *M*. This gives an obstruction in the Pontryagin classes to the existence of nondegenerate umbilic foliations by hypersurfaces. To the best of the author’s knowledge, this is the first of such an obstruction using Pontryagin classes. However, some nonexistence results are known. In [16] it is shown that odd-dimensional spheres do not admit umbilic foliations with an integrable normal bundle. More generally, in [28] some nonexistence results are given for foliations by umbilic hypersurfaces for compact Riemannian manifolds of constant sectional curvature. However, both [16] and [28] do not use methods based on characteristic classes. We conclude the paper with the following obstruction to the existence of foliations by nondegenerate umbilic hypersurfaces.

#### Theorem 3.23

(Pontryagin class obstruction for nondegenerate umbilic foliations). Let (*M*, *g*) be a 4*k*-dimensional pseudo-Riemannian manifold. If there exists a foliation of (*M*, *g*) by nondegenerate umbilic hypersurfaces. Then for all multi-indices α with |α|=k, we have $p_{\alpha }(M) = 0$.

Conversely, if *M* is compact and orientable and there exists a multi-index α with |α|=k such that $p_{\alpha }(M)\ne 0$, then there exist no pseudo-Riemannian metric on *M* and codimension 1 foliation of *M* for which all leaves of the foliation are nondegenerate and umbilic. □

## Data Availability

Data sharing not applicable to this article as no datasets were generated or analyzed during the current study.

## Appendix A Proof of Theorem 2.18

In this appendix, we will provide a proof of Theorem 2.18. For which we first introduce the following useful notation. Recall that $[n] = \{1, \ldots , n\}$. We denote by $\mathcal {P}([n])$ the powerset of [*n*] and by $\mathcal {P}([n])_k$ the set of subsets of [*n*] with *k* elements. If we consider a multi-index $I = (i_1, \ldots , i_k)\in [n]^k$ and $\sigma \in S_I$ is a permutation of *I*, then we denote by $I_{\sigma } = (\sigma (i_1), \ldots , \sigma (i_k))$ the permuted multi-index. Also, if $T \in (V^*)^{\otimes k}$ is a *k*-multilinear map and $\sigma \in S_k$ is a permutation on *k* elements. We define $T_\sigma \in (V^*)^{\otimes k}$ by

$$ T_\sigma (x_1,\ldots , x_k) = T(x_{\sigma (1)},\ldots , x_{\sigma (k)}), $$

for all $$x_1, \ldots , x_k \in V$$.

If *e* is an orthonormal basis for *V*, then we define the *causal character signs* of the basis vectors by $\varepsilon _i = g(e_i, e_i) \in \{\pm 1\}$. More generally, a multi-index $I = (i_1, \ldots , i_k)\subseteq [n]$ of length *k* defines a *k*-vector $e_I = e_{i_1}\wedge \cdots \wedge e_{i_k}$. We define the causal character sign of $e_I$ to be $\varepsilon _I = \langle e_I, e_I\rangle _g$. In other words,

$$ \varepsilon _I = \prod _{i\in I}\varepsilon _i, $$

if *I* has no repeating indices, and $\varepsilon _I = 0$ otherwise. Note this does not depend on the order of $(i_1, \ldots , i_k)$, i.e., for all $\sigma \in S_I$, we have $\varepsilon _I = \varepsilon _{I_\sigma }$.

Choosing a basis $e = (e_1, \ldots , e_n)$ for *V* and an algebraic curvature tensor $C\in \mathcal {C}(V)$, we obtain the 2-forms  defined by

A.1

which can be chosen in such a way that . It is easy to show that the 2-forms  of the curvature operator as defined in (A.1) and the 2-forms of the curvature matrix as defined in (2.7) are related via

A.2

The outline of the proof is as follows. In Lemma A.1, we first express the higher curvature operators of *C* in terms of the 2-forms introduced in (A.1). This way, we can also rewrite the 4*k*-form $F_{2k}(\hat{C}^{*k}, \hat{C}^{*k})$ from Theorem 2.18 in terms of the 2-forms from (A.1). Then in Lemma A.2, we will give an explicit expression for the polynomials $\sigma _k^{(n)}$. Using the obtained formulas, we can give an explicit expression for the Pontryagin form $\varpi _k(C)$, which after some algebraic manipulations is seen to equal the right-hand side of Theorem 2.18

### Lemma A.1

For all $$1\le k\le \lfloor \frac{n}{2} \rfloor $$,

A.3

### Proof

This follows directly by induction. The base case (k=1) follows by definition of the 2-forms of the curvature operator in (A.1) and the induction step is performed by applying (2.4) to $A = \hat{C}^{*k}$ and $B = \hat{C}$. □

We proceed to give an explicit formula for the polynomials $\sigma ^{(n)}_k$.

### Lemma A.2

Let  be a square matrix. Then

A.4

where $\operatorname {sgn}(I;J)$ equals $\operatorname {sgn}(\sigma )$ if *I* and *J* have no repeated elements and $J = I_\sigma$, and equals 0 otherwise.

### Proof

By definition of the determinant and the polynomials $\sigma ^{(n)}_k$, we have

where δ denotes the Kronecker delta. Note that

A.5

so a summand in (A.5) vanishes unless σ fixes [n]\I. The resulting expression reads

A.6

We now re-index the sum in the right-hand side of (A.6) in order to isolate the powers of *t*, keeping in mind that a summand in (A.6) vanishes unless σ fixes [n]\I. Consider the sets

$$ A_1:= \{(\sigma , I) \in S_n\times \mathcal {P}([n]): \sigma \, {\text {fixes}}\, [n]\setminus I\} \qquad {\text {and}} \qquad A_2 = \coprod _{I \subseteq [n]}S_I. $$

On these sets consider the functions $f:A_1\rightarrow A_2$ defined by $(\sigma , I)\mapsto (I, {\sigma }\Big |_{I})$ and $g:A_2\rightarrow A_1$ defined by $(I, \tau ) \mapsto (\tilde{\tau }, I)$, where $\tilde{\tau }$ is the extension of τ to [*n*] by declaring $\tilde{\tau }$ to be the identity on [n]\I. It is easy to see that *f* and *g* are mutual inverses, and therefore bijections. Using these bijections and the fact that if σ fixes [n]\I, then $\operatorname {sgn}(\sigma ) = \operatorname {sgn}({\sigma }\Big |_{I})$, we see that

Comparing coefficients of $t^k$, we see that

A.7

A.8

The factor $\frac{1}{k!}$ in (A.8) compensates for the fact that for a fixed $I_0\subseteq [n]$ of length *k*, in (A.7) only on the second index of the matrix entries, all possible permutations of $I_0$ are being summed over, whereas in (A.8) also on the first index of the matrix entries, all *k*! different permutations of $I_0$ are being summed over. □

We now combine our results from Lemmas A.1 and A.2 to prove Theorem 2.18.

### Proof of Theorem 2.18

Using Lemma A.2, we find that

A.9

Note that the summands in (A.9) are only nonvanishing if the multi-index *J* is a permutation of the multi-index *I* and both have no repeating indices, for $\operatorname {sgn}(I;J) = 0$ otherwise. This means *I* and *J* define the same subset $A \in \mathcal {P}([n])_{2k}$ and when *A* is considered as a multi-index of length 2*k* with the natural increasing order, there exist unique permutations $\sigma , \tau \in S_{A}$ such that $I = A_{\sigma }$ and $J = A_{\tau }$. Conversely, any subset A⊆[n] of length 2*k* and permutations $\sigma , \tau \in S_{A}$ define such *I* and *J* uniquely. So the sum in (A.9) can instead be taking over such *A*, σ and τ. It follows that $\varepsilon _I = \varepsilon _{A}$ and

$$ \operatorname {sgn}(I;J) = \operatorname {sgn}(\tau \sigma ^{-1}) = \operatorname {sgn}(\tau )\operatorname {sgn}(\sigma ). $$

Combining this, we see that

A.10

A.11

where (A.10) follows by Chern’s formula on generalized Pfaffian functions [13, Ch. 2].

On the other hand, if *I* and *J* are two multi-indices of elements in [*n*] of length 2*k*, then it follows that

A.12 $$\begin{aligned} \langle e_I, e_J \rangle _g&= \det [(g(e_{i_a}, e_{j_b}))_{a, b\in [2k]}] = \det [(\varepsilon _{i_a}\delta _{i_a, j_b})_{a, b\in [2k]}] \nonumber \\&= \prod _{i\in I}\varepsilon _i\cdot \det [(\delta _{i_a, j_b})_{a, b\in [2k]}] = \varepsilon _I\operatorname {sgn}(I;J). \end{aligned}$$

Computing $F_{2k}(\hat{C}^{*k}, \hat{C}^{*k})$ using Lemma A.1 and (A.12), we find that

A.13

where the final equality follows from the definition of the wedge product on differential forms. Again using the fact that the terms in (A.13) are only nonvanishing if the multi-index *J* is a permutation of the multi-index *I* and both do not have a repeating index and that in this case *I* and *J* uniquely define a set $A\in \mathcal {P}([n])_{2k}$ and permutations $\sigma , \tau \in S_A$ such that $I = A_{\sigma }$ and $J= A_{\tau }$, we can rewrite (A.13) as

A.14

The result follows from combining (A.11) and (A.14). □
